# Homeogene *emx1* is required for nephron distal segment development in zebrafish

**DOI:** 10.1038/s41598-018-36061-4

**Published:** 2018-12-21

**Authors:** Elvin E. Morales, Nicole Handa, Bridgette E. Drummond, Joseph M. Chambers, Amanda N. Marra, Amanda Addiego, Rebecca A. Wingert

**Affiliations:** Department of Biological Sciences, Center for Stem Cells and Regenerative Medicine, Center for Zebrafish Research, University of Notre Dame, Notre Dame, IN 46556 USA

## Abstract

Vertebrate kidneys contain nephron functional units where specialized epithelial cell types are organized into segments with discrete physiological roles. Many gaps remain in our understanding of how segment regions develop. Here, we report that the transcription factor *empty spiracles homeobox gene 1* (*emx1*) is a novel nephron segment regulator during embryonic kidney development in zebrafish. *emx1* loss of function altered the domains of distal segments without changes in cell turnover or traits like size and morphology, indicating that *emx1* directs distal segment fates during nephrogenesis. In exploring how *emx1* influences nephron patterning, we found that retinoic acid (RA), a morphogen that induces proximal and represses distal segments, negatively regulates *emx1* expression. Next, through a series of genetic studies, we found that *emx1* acts downstream of a cascade involving *mecom* and *tbx2b*, which encode essential distal segment transcription factors. Finally, we determined that *emx1* regulates the expression domains of *irx3b* and *irx1a* to control distal segmentation, and *sim1a* to control corpuscle of Stannius formation. Taken together, our work reveals for the first time that *emx1* is a key component of the pronephros segmentation network, which has implications for understanding the genetic regulatory cascades that orchestrate vertebrate nephron patterning.

## Introduction

Ontogeny of the vertebrate kidney is a dynamic process involving the sequential formation and regression of multiple structures from the intermediate mesoderm (IM) during embryogenesis^1^. These developmental events can encompass up to three renal forms, termed the pronephros, mesonephros, and metanephros, that share a composition of nephron subunits in various anatomical arrangements^1^. In mammals, for example, the first two kidneys are transient structures where only the second functions during gestation, and the third form becomes the adult organ^2,3^. In lower vertebrates, such as fish, the pronephros is both transient as well as functional, and adults retain a mesonephros that develops during juvenile stages^2,3^. Nephron units serve to filter the blood, and then modify this excretory fluid to retain essential nutrients and regulate water balance. Each nephron has discrete segments of epithelial cells specialized for particular secretory and absorptive tasks. While segment organization is broadly conserved across vertebrate kidney forms, differences enable functionalities such as enhanced water retention in terrestrial species^4^. The pathways that direct segment regionalization in developing nephrons across kidney forms are beginning to be understood, and recent advances have revealed insights into the mechanisms of segment pattern formation, growth, and terminal differentiation in several vertebrate species^5–12^.

The zebrafish is a useful genetic model to study organogenesis^13^, and is particularly amenable to renal development and disease studies because the embryonic pronephros has a simple anatomy of two nephrons that have a segment composition like other vertebrates^14–16^. The pronephros develops rapidly, forming segments by 24 hours post fertilization (hpf), and the nephrons are also linear at this time, facilitating the precise assessment of segmentation events^17^ and subsequent physiological functions^18^. The nephrons share a single blood-filtering glomerulus, and their epithelial tubules have two proximal segments (proximal convoluted tubule, PCT; proximal straight tubule, PST) and two distal segments (distal early, DE; distal late, DL) that are closely associated with a gland known as the corpuscle of Stannius (CS), followed by a pronephric duct (PD)^19^.

To date, several studies have identified essential signaling molecules and transcription factors that mitigate pronephros segmentation, which has been speculated to offer a blueprint for conserved segmentation mechanisms across kidney forms^5^. During distal pronephros development, the Iroquois homeodomain transcription factor *Iroquois homeobox 3b* (*irx3b*)/*Irx3* specifies the DE segment, an aspect shared between zebrafish and *Xenopus*, respectively^20,21^. In zebrafish, patterning of the DL segment is reliant on the zinc finger transcription factor *MDS1 and EVI1 complex locus* (*mecom*)^22^ as well as the T-box transcription factors *T-box 2a* (*tbx2a*) and *T-box 2b* (*tbx2b*)^23^, though the relationships between these key factors has not yet been established. A crucial upstream effector of proximal and distal cell fate decisions during the process of zebrafish pronephros segmentation is retinoic acid (RA) signaling^19^. RA promotes proximal segment identities and inhibits distal ones through patterning events that transpire during pre-gastrula and gastrula stages^19,20,24^ and lead to alterations in the expression domains of key transcription factors that include *irx3b*^20^, *mecom*^22^, *tbx2a/b*^23^, *sim1a*^25^, and *etv5a*^26^. The spatial expression domains of several other transcription factors in the renal progenitors are also altered due to the abrogation of RA biosynthesis or RA receptor signaling^20^, though their functional contribution(s) to nephrogenesis have yet to be assigned.

*emx1* encodes a homeodomain transcription factor that is expressed in renal progenitors during early stages of zebrafish pronephros development^27^, and numbers among those genes that have been implicated to act downstream of RA signaling during nephron segmentation^20^. *emx1* is related to the fruitfly gene *empty spiracles* (*ems*)^28^, which controls the formation of anterior head segments and the development of the olfactory system^29,30^. In zebrafish and rodents, *emx1/Emx1* is expressed in the developing brain^27,31–33^, and *Emx1* deficient mice evince developmental defects in the forebrain including disruption of corpus callosum formation^34,35^. Interestingly, microarray data curated on the Genitourinary Development Molecular Anatomy Project (GUDMAP) localized murine *Emx1* transcripts in the metanephros to the renal vesicle at stage E12.5, then to S-shaped body structures, proximal tubules and loop of Henle anlage at E15.5, which may indicate roles in nephrogenesis^36^. Also, *Emx1* expression was detected in the ureteric bud at E11.5 and ureteric tips at E15.5, suggesting involvement with collecting system formation^36^. Despite their intriguing expression patterns during vertebrate nephrogenesis, however, the function of *emx1/Emx1 *during nephron ontogeny has not been examined.

Here, we establish several roles for *emx1* during nephron distal segment patterning in the zebrafish embryo kidney. Using loss of function techniques, we found that *emx1* is necessary to pattern the neighboring DE and DL segment domains as well as to repress CS fate. We ascertained that RA signaling negatively regulates the domain of *emx1* expression in the pronephros. Through genetic studies, we then determined that *emx1* acts downstream of the distal patterning factors *mecom* and *tbx2b*, which we show here to comprise a cascade that promotes DL formation. Finally, we show that *emx1* is necessary to restrict the expression domains of *irx3b, irx1a* and *sim1a*, which are known to mitigate DE and CS development, respectively. Taken together, these results provide new evidence that *emx1* is a critical component of regulating segmentation in the pronephros, and suggest for the first time that *emx1* has key roles in vertebrate mesoderm development. These findings have implications for understanding the basis of congenital anomalies of the kidney and urinary tract (CAKUT) and current approaches in regenerative medicine such as renal organoids.

## Results

### *emx1* expression is dynamic in renal progenitors and then localizes to the DL segment

**Figure 1:**
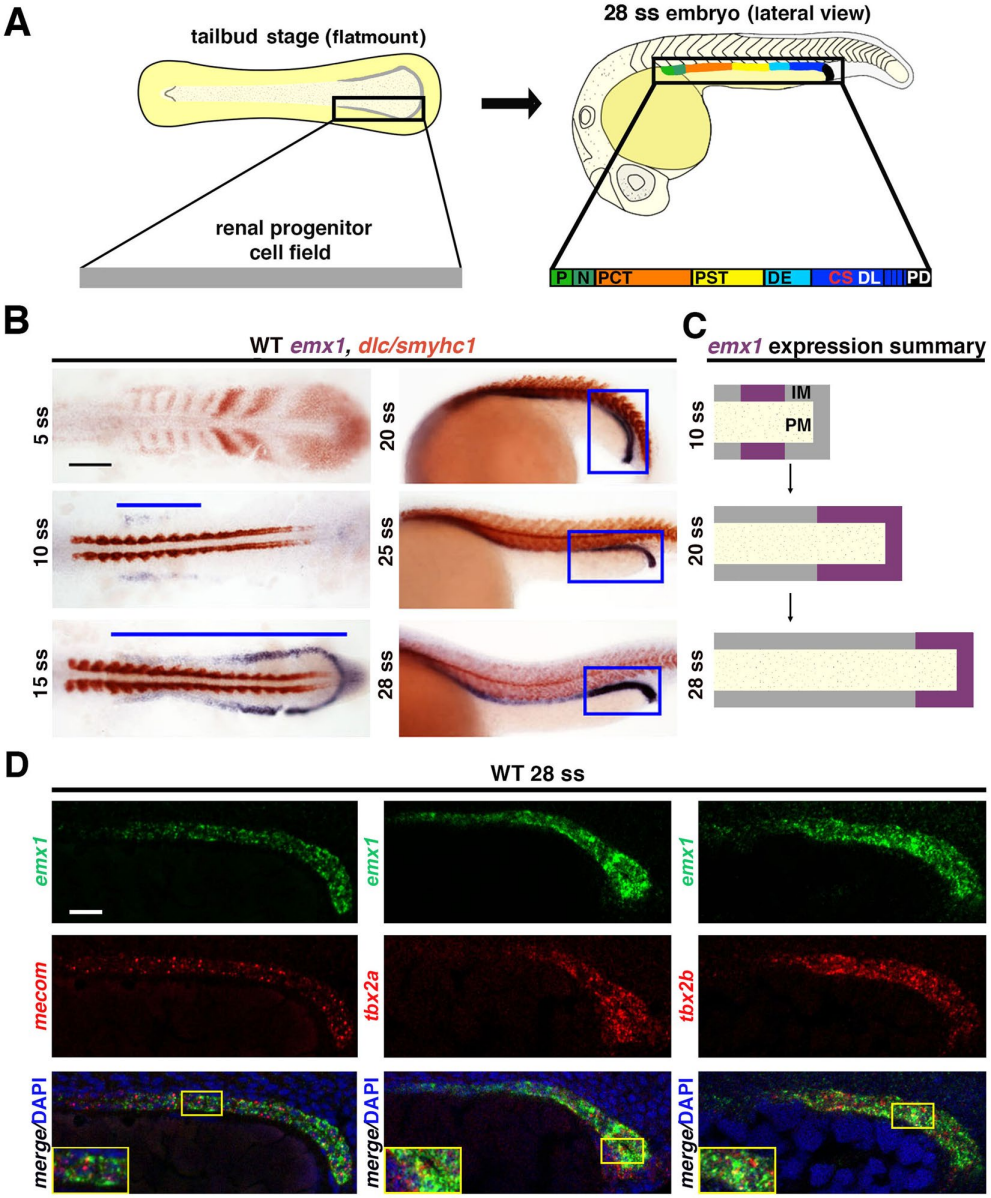
*emx1* is dynamically expressed during zebrafish pronephros ontogeny. (**A)** The renal progenitor field is patterned into a segmented pronephros with the following 8 regions: P, podocytes; N, neck; PCT, proximal convoluted tubule; PST, proximal straight tubule; DE, distal early; CS, corpuscle of Stannius; DL, distal late; PD, pronephric duct. **(B)** WISH in WT embryos for *emx1* (purple) and *smyhc1* (red) between 5 ss and 28 ss. Blue lines and boxes indicate domains of pronephros expression. Scale bar = 100 μm. **(C)** Summary of the *emx1* expression domain during nephrogenesis, where IM designates the intermediate mesoderm renal progenitors, and PM designates paraxial mesoderm. **(D)** FISH analysis of *emx1*, *mecom*, *tbx2a*, and *tbx2b* in the distal pronephros. Yellow boxes demarcate areas shown in insets. Scale bar = 50 μm.

The zebrafish pronephros arises from bilateral stripes of IM precursors that develop into a pair of parallel nephrons by the 28 somite stage (ss) (Fig. 1A)^17^. This process involves a mesenchymal to epithelial transition of the renal progenitors^37,38^ along with their pattern formation into a series of at least 8 discrete segment domains, wherein the segments contain populations of like transporter cells that in some regions are intermingled with a discrete subset of multiciliated cells^39^. Prior studies have reported the localization of *emx1* transcripts between the 15 ss and 28 ss in a spatial arrangement consistent with the IM and pronephros territories^20,27^.

To more closely examine the spatiotemporal location of *emx1* transcripts during mesoderm development, we performed a series of double whole mount *in situ* hybridization (WISH) experiments over a developmental time course between the tailbud stage and the 28 ss. We used a combination of riboprobes to detect *emx1* in the IM relative to the adjacent paraxial mesoderm (PM) territory using the marker *deltaC* (*dlc*), which enables visualization of the early somites, or *slow myosin heavy chain 1* (*smyhc1*), which marks somites at later stages. *emx1* expression was first detected adjacent to the somites at the 10 ss in a subset of the renal progenitor field, while transcripts were present throughout the entire renal progenitor field by the 15 ss (Fig. 1B). Between the 20–28 ss, *emx1* transcripts localized to the presumptive distal segment territories and became restricted to the putative DL domain by the 28 ss (Fig. 1B). These data indicate that the *emx1* expression domain is dynamic within the developing pronephros (schematized in Fig. 1C), similar to many other genes^20^.

To define further how the domain of *emx1* expression aligned with the DL segment, we performed double fluorescent *in situ* hybridization (FISH) expression studies with confocal imaging at the 28 ss with several markers of this nephron population. Prior studies have localized *mecom* transcripts to the DL segment^22^ and demonstrated the co-localization of *tbx2a* and *tbx2b* with the DL specific marker *solute carrier family 12* (*sodium/chloride transporter*), *member 3* (*slc12a3*)^23^. We found that *emx1* and *mecom* transcripts co-localized in the DL segment, and that *emx1* transcripts also co-localized with *tbx2a* and *tbx2b* (Fig. 1D). These results support the conclusion that the DL segment contains a common populace of *emx1*, *mecom*, and *tbx2a/b* expressing cells. This suggested that *emx1* may have roles in regulating DL development, and potentially other pronephros segments as well in light of its dynamic expression pattern in renal progenitors.

### *emx1* deficient embryos have altered distal tubule segments and CS development

We conducted loss of function studies to assess the role of *emx1* in pronephros development. To create models of *emx1* deficiency, we first performed knockdown studies with a morpholino combination that was designed to interfere with the splice donor site of exon 1 and the splice acceptor site of exon 2 (Fig. S1A). Following microinjection of the splice targeting combination in fertilized wild-type (WT) embryos at the 1-cell stage, we collected the embryos at the 28 ss and performed RNA extraction. Through RT-PCR analysis we determined that the splice morpholino combination led to production of an incorrectly processed *emx1* transcript, with an in-frame premature stop codon due to the inclusion of intronic sequence (Fig. S1A,B). As the predicted Emx1 protein encoded by this transcript is truncated and entirely lacks the homeodomain sequence, we concluded that transcriptional activity is likely abrogated in proteins produced from these altered transcripts.

**Figure 2:**
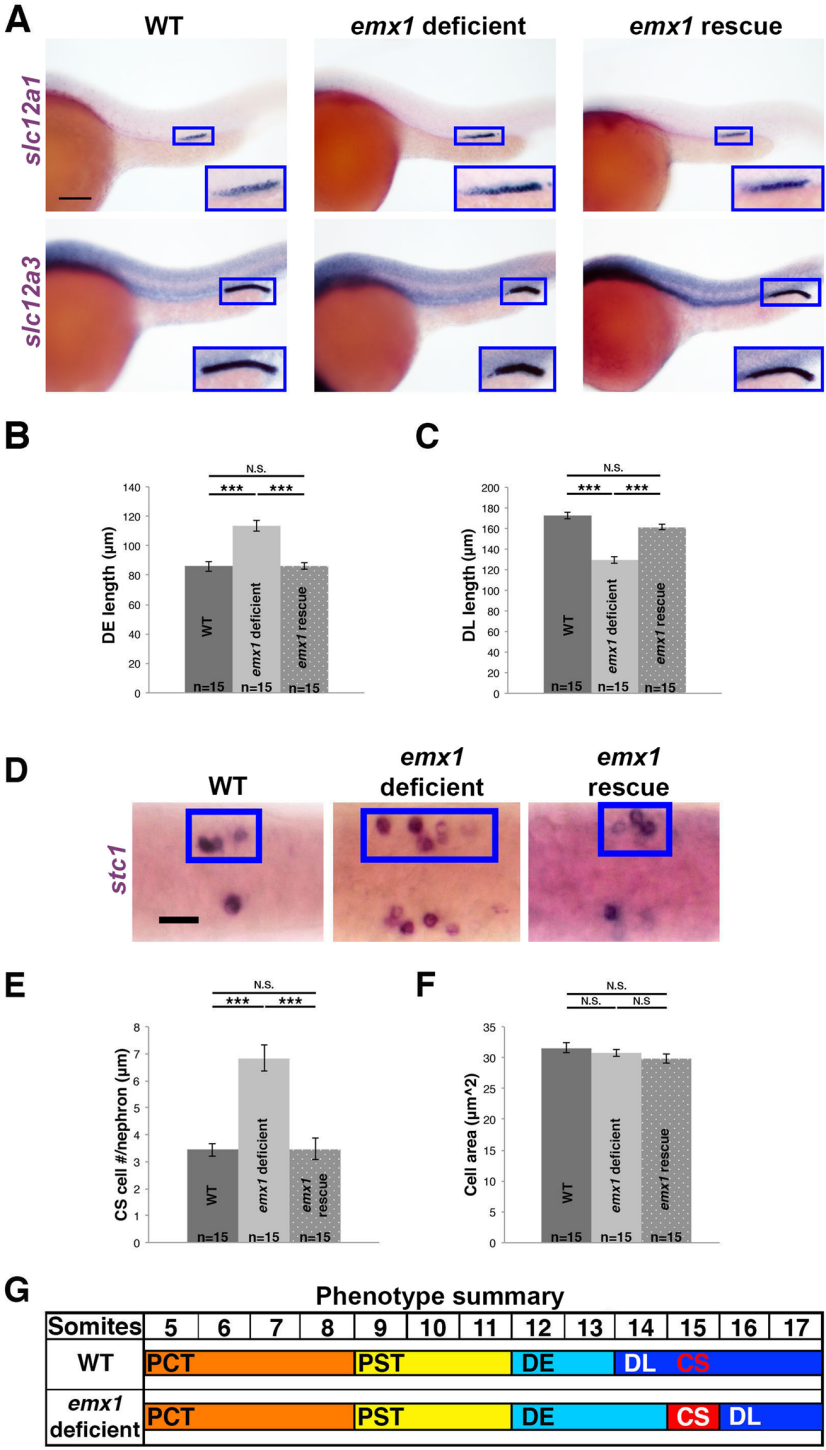
*emx1* deficient embryos exhibit a DE expansion, DL reduction and an expansion of the associated CS gland. **(A)** WISH analysis (lateral view) of 28 ss WT, *emx1* deficient, and *emx1* deficient embryos co-injected with *emx1* mRNA (*emx1* rescue) for the distal segment markers (purple): *slc12a1* for the DE, and *slc12a3* for the DL. Blue boxes demarcate areas of pronephros gene expression. Scale bar = 100 μm. **(B**,**C)** Average segment lengths of the DE and DL for WT, *emx1* deficient, and *emx1* rescue embryos. **(D)** WISH panel (dorsal view) of 28 ss WT, *emx1* deficient, and *emx1* rescue embryos for the CS marker *stc1* (purple). Blue boxes demarcate a single CS territory while the contralateral side is unlabeled. Scale bar = 20 μm. Quantification of *stc1*^+^ cell number **(E)** and size **(F)** of WT, *emx1* deficient, and rescue embryos. ***p < 0.001; N.S. = not significant. Error bars indicate standard error. **(G)** Summary of pronephros phenotype in *emx1* deficient embryos with respect to the axial location of somites.

To explore the roles of *emx1* in renal development, we assessed the expression domains of segment-specific markers using WISH in in *emx1* deficient embryos compared to WT controls at the 28 ss. *emx1* deficiency was associated with alterations in formation of both the DE and DL segment domains (Fig. 2A–C). The DE segment, marked by *solute carrier family 12* (*sodium/potassium/chloride transporter*)*, member 1* (*slc12a1*) was expanded in *emx1* deficient embryos (Fig. 2A) and measurement of absolute segment length revealed a statistically significant increase in the DE domain compared to WT control embryos (Fig. 2B). In contrast, the DL segment, marked by *slc12a3* was reduced in *emx1* deficient embryos (Fig. 2A) and measurement of absolute segment length revealed a statistically significant decrease in the DL compared to WT control embryos (Fig. 2C). Evaluation of the other tubule nephron segments with WISH revealed normal spatial expression domains of PCT, PST, and PD markers along with normal domains of both pan-proximal (PCT, PST) and pan-distal markers (DE, DL) compared to WT sibling control embryos (Figs S1C–J, S2). Given the alterations in the distal tubule segments, we also examined development of the CS lineage, which emerges from the IM in the vicinity occupied by DE and DL precursors^19,40,41^. Interestingly, *emx1* deficient embryos had an increased expression domain of the CS specific marker *stanniocalcin 1 (stc1)* (Fig. 2D). Quantification of *stc1*^+^ expressing cells revealed that there was a significant increase in the number of CS cells associated with each nephron (Fig. 2E), while the area of individual cells was unchanged (Fig. 2F).

Next, we obtained a published *emx1* morpholino that blocks the translational start site^42^, and examined nephron segmentation and CS development at the 28 ss (Figs S3, S4). Analysis of these *emx1* deficient embryos showed similar phenotypes, with of an expanded DE segment, shortened DL segment and expanded CS populace (Figs S3, S4). The similarities in pronephros and CS phenotypes in these distinct models of *emx1* deficiency strengthened our conclusion that Emx1 is necessary for formation of the DE and DL segments as well as the CS.

To further discern whether these phenotypes were caused by the loss of Emx1 activity or possibly influenced by off-target effects, we performed rescue studies in *emx1* deficient embryos to test whether the expression of *emx1* transcripts that encoded the full-length mature mRNA sequence would be sufficient to restore pronephros and CS development. Following *in vitro* mRNA synthesis and purification, we co-injected *emx1* transcripts with the splice morpholino combination at the 1 cell stage and fixed embryos at the 28 ss to examine segment domains using WISH. We observed a normal DE, DL and CS in *emx1* deficient embryos that were co-injected with *emx1* transcripts (Fig. 2A–F). Taken together, these results led us to conclude that the loss of Emx1 specifically alters the balance of distal segment fates, leading to increases in the DE segment and CS lineages along with a concomitant reduction in the DL segment (Fig. 2G).

### *emx1* deficiency does not alter nephron segment traits of cell turnover, size or arrangement

**Figure 3:**
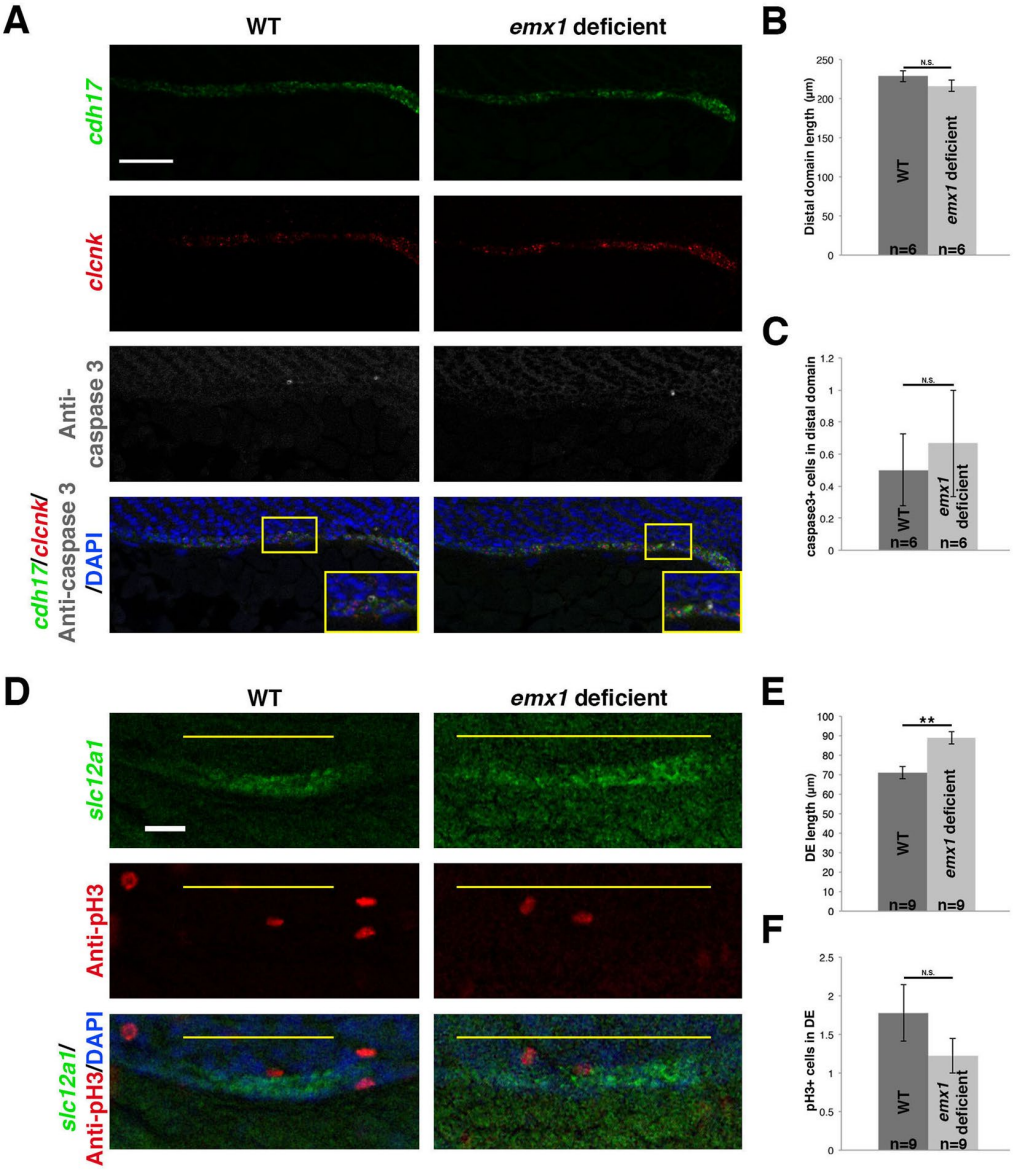
*emx1* deficiency is not associated with changes in cell death or proliferation within the pronephros. **(A)** Confocal imaging of double FISH analysis (lateral view) of 28 ss WT and *emx1* deficient embryos for the renal epithelial cell marker *cdh17* (green), the pan-distal segment marker *clcnk* (red), combined with protein localization of active caspase-3 (grey) and DAPI staining (blue) through IHC. Yellow boxes demarcate areas shown in insets. Scale bar = 50 μm. (**B**,**C**) Quantification of caspase-3^+^ cell number and *clcnk* domain. (**D**) Confocal imaging of FISH analysis (lateral view) of 28 ss WT and *emx1* deficient embryos for the DE marker *slc12a1* (green), with protein localization of pH3 (red) and DAPI (blue) using IF. Yellow lines demarcate the DE domain. Scale bar = 20 μm. **(E**,**F**) Quantification of pH3^+^ cells in the DE and average DE length. **p < 0.01; N.S. = not significant. Error bars indicate standard error.

We next determined if *emx1* affected the balance of distal segment fates by influencing the cellular dynamics of populations in this developing region, as early segment size in the zebrafish pronephros is affected by proliferation^43^. As changes in the distal segment domains of *emx1* deficient embryos were first observed between the 26–28 ss (Figs 2 and S2–S4, data not shown), we examined cell dynamics at this time. Using acridine orange staining, we observed no difference in the number of cells undergoing apoptosis within the distal tubule or its neighboring tissues at the 28 ss in *emx1* deficient embryos compared to WT controls (Fig. S5). Elevated numbers of acridine orange^+^ cells were detected within the central nervous system of *emx1* deficient embryos compared to WT controls (Fig. S5), which may reflect *emx1* function(s) in neuronal development. To further assess cell death, we performed whole mount immunofluorescence to detect activated endogenous Caspase-3 in combination with FISH to examine the distal pronephros based on expression of the pan-tubule marker *cadherin 17 (cdh17)* and the pan-distal segment/CS marker *chloride channel K (clcnk)*. As in our prior WISH studies, the absolute length of the pan-distal segment domain was unchanged in *emx1* deficient embryos, and within this domain, there was no significant difference in the number of Caspase-3^+^ cells compared to WT control embryos (Fig. 3A–C). These results indicate that the reduced DL segment population in *emx1* deficient embryos does not originate with enhanced cell death within this region.

Subsequently, we examined cell proliferation by whole mount immunofluorescence (IF). For this, we detected phospho-Histone H3 (pH3) staining in combination with FISH to identify the DE segment populace based on their expression of *slc12a1* transcripts. *emx1* deficient embryos formed an expanded *slc12a1*^+^ domain, as observed previously, and quantification of the pH3^+^ cell number within the DE showed no significant difference to WT control embryos at the 28 ss (Fig. 3D–F). This indicates that an alteration in cell proliferation within the DE segment does not underlie the significant expansion of this domain in *emx1* deficient embryos. Further, alterations in pH3^+^ number were not observed in the neighboring regions either (data not shown), consistent with the conclusion that the CS lineage was also not proliferating at an increased rate in *emx1* deficient embryos. Thus, our results indicate that Emx1 does not influence cellular turnover in the developing pronephros or CS that leads to segmentation changes.

**Figure 4:**
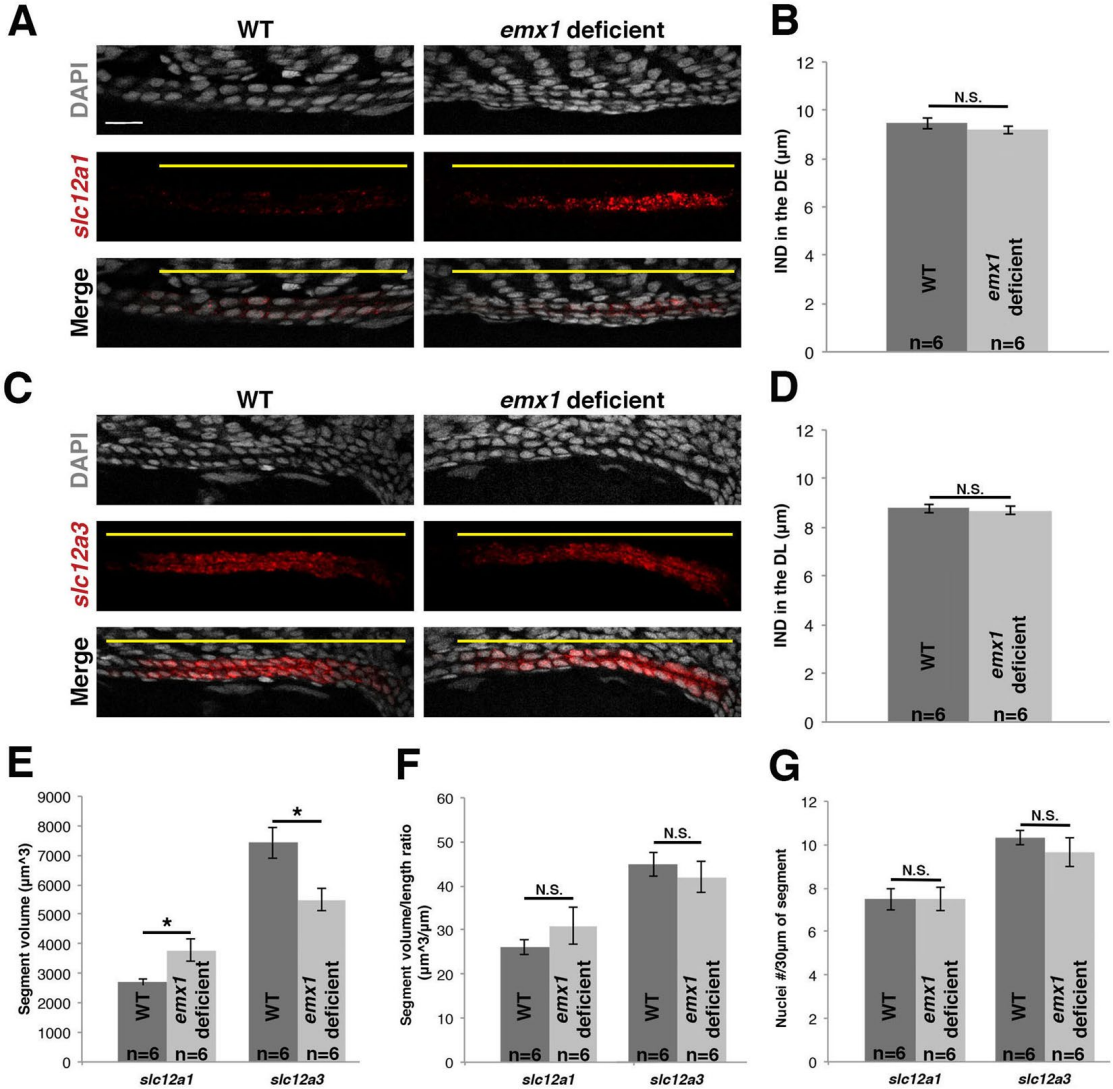
Spatial arrangement and size of renal cells in the DE and DL are unaffected by *emx1* deficiency. (**A**,**C**) Confocal imaging of FISH analysis (lateral view) of 28 ss WT and *emx1* deficient embryos for the DE and DL segment makers *slc12a1* and *slc12a3* (red), respectively, with DAPI staining (grey). Yellow lines demarcate segment domains. Scale bar = 20 μm. **(B**,**D)** Quantification of internuclear distance of DE and DL segment cells in WT and *emx1* deficient embryos. Quantification of tubule volume **(E)**, segment volume to segment length ratio **(F)**, and the number of nuclei per 30 μm of segment in WT and *emx1* deficient DE and DL segments **(G)**. *<0.05; N.S. = not significant. Error bars represent standard error.

A remaining possibility that could explain the expanded DE and reduced DL segment sizes would be alterations in the size or arrangement of individual cells in these populations. Therefore, we performed whole mount FISH and confocal imaging to examine these features. The DE and DL segment populations were again visualized based on their expression of *slc12a1* and *slc12a3*, respectively, and cell nuclei were labeled using DAPI. First, we measured the internuclear distance (IND) of cells in each segment. Within the expanded DE of *emx1* deficient embryos (Fig. 4A), there was no change in the IND compared to WT embryos (Fig. 4B). Likewise, within the reduced DL segment of *emx1* deficient embryos (Fig. 4C), there was no change in the IND compared to WT embryos (Fig. 4D). We next measured segment volume in *emx1* deficient embryos and WT controls. Consistent with the alterations in segment length, the DE was significantly higher in volume, and the DL was significantly reduced in volume, in *emx1* deficient embryos (Fig. 4E). However, the segment volume to length ratio or the number of nuclei in a given area was not significantly different between *emx1* deficient and WT control embryos in the DE or DL (Fig. 4F,G). These analyses indicate that the expanded DE and reduced DL are not related to changes in cell dimension or arrangement differences. As changes in cellular turnover were also not detected in *emx1* deficient embryos, we concluded that the alterations in the DE and DL segments reflect changes in cell fates of the renal progenitors.

### RA signaling negatively regulates *emx1* expression within the developing pronephros

Since our results indicated that Emx1 is required to regulate distal segment patterning by influencing cell fate of the nephron precursors, we next sought to determine the relationship of *emx1* with known patterning factors that regulate fate choice in the IM. RA signaling acts to regionalize the renal progenitors into proximal and distal domains along the embryonic trunk^19^. Elevating bioactive retinoid levels by treatment with exogenous all-trans RA expands proximal segment domains and reduces distal segments; conversely, genetic mutations in the RA biosynthetic enzyme encoded by *aldehyde dehydrogenase 1 family, member A2* (*aldh1a2*) or blocking RA production with the Aldh inhibitor 4-diethylaminobenzaldehyde (DEAB) expands distal segments and reduces proximal segments^19,20^. Alterations in the levels of RA have dramatic consequences for nephron patterning due to changes in the spatiotemporal expression of essential transcription factors. Thus, we hypothesized that RA was likely to regulate *emx1* expression in the zebrafish pronephros.

**Figure 5:**
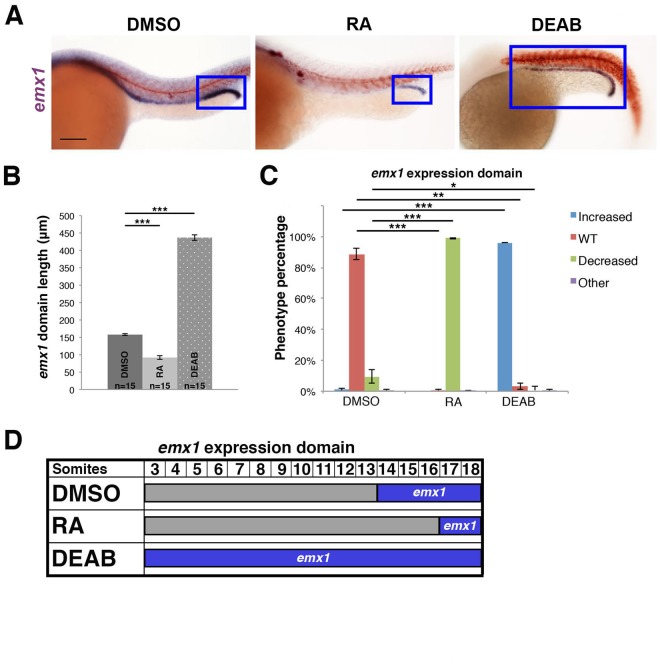
*emx1* expression within the pronephros is negatively regulated by RA signaling. **(A)** WISH panel (lateral view) of *emx1* expression in 28 ss embryos treated with DMSO vehicle, all-trans RA, or DEAB. Blue boxes demarcate domains of *emx1* expression in the pronephros. Scale bar = 100 μm. **(B)** Quantification of the *emx1* expression domain in DMSO, RA, and DEAB treated embryos. **(C)** Quantification of phenotypes, categorized as increased, WT, decreased, or other, where the latter include those developmentally delayed or too dysmorphic to measure. **(D)** Summary of the *emx1* expression domain in treatment groups. *p < 0.05; **p < 0.01; ***p < 0.001. N.S. = not significant. Error bars represent standard error.

To test this, WT embryos were treated with all-trans RA, DEAB, or dimethyl sulfoxide (DMSO) as a vehicle control between 60% epiboly until 16.5 hpf when the solutions were removed and replaced with E3 media. The embryos were fixed when they reached the 28 ss, and double WISH was performed to assess the *emx1* expression domain within the pronephros with respect to the axial location of somites along the trunk, where the latter was visualized based on *smyhc1* expression. Exogenous RA reduced the *emx1* expression domain within the pronephros (Fig. 5). Conversely, DEAB treatment expanded the *emx1* domain in the nephrons (Fig. 5). These alterations in e*mx1* domain parallel alterations in the domain of *slc12a3* expression, which marks the DL (Fig. S6). These results are consistent with the known effects of RA to promote proximal segment fates and inhibit distal segment fates, and suggest that RA signaling in renal progenitors negatively regulates the spatial domain of *emx1* expression.

RA signaling works by RA binding to the RA receptor (RAR), which interacts with DNA as a heterodimer with the retinoid X receptor (RXR) on regions known as Retinoic Acid Response Elements (RAREs). The consensus sequence for these RAREs is a direct repeat of the following motif: 5′- PuG(G/T)TCA spaced by 1, 2 or 5 base pairs^44^. Thus, we examined the promoter region of *emx1* for the presence of RAREs to explore the possibility that RA directly regulates *emx1*. Interestingly, no RAREs were found within the *emx1* promoter (data not shown), suggesting that RA has an indirect relationship with *emx1*.

### A hierarchy of the transcription factors encoded by *mecom*, *tbx2b*, and *emx1* controls pattern formation of the DL nephron segment

In the zebrafish pronephros, DL segment development is reliant on activity of the transcription factors encoded by *mecom* and *tbx2a/b*, where loss of either *mecom* or *tbx2a/b* function results in a shorter DL segment^22,23^. Further, these genes act downstream of RA and are both negatively regulated by RA during nephron ontogeny^22,23^, similar to *emx1*. Given our results that *emx1* is necessary for DL development, and causes a nearly identical DL phenotype compared to *mecom* or *tbx2a/b* loss of function, we next sought to delineate the relationships between these transcription factors and *emx1*.

**Figure 6:**
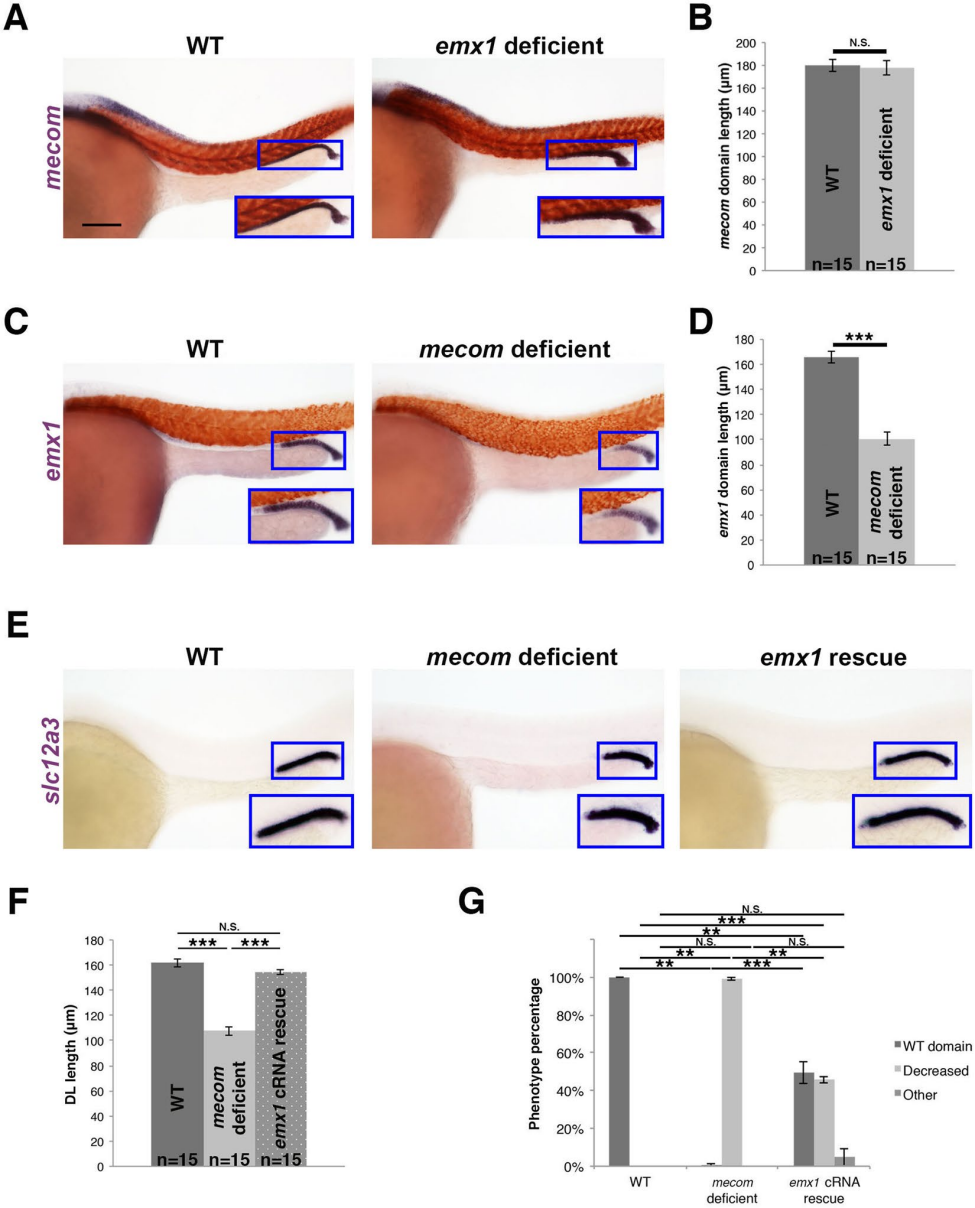
*mecom* acts upstream of *emx1* to regulate DL segment development. **(A)** WISH (lateral view) of 28 ss WT and *emx1* deficient embryos for *mecom* (purple) and *smyhc1* (red) expression. **(B)** Quantification of *mecom* domain length. **(C)** WISH of 28 ss WT and *mecom* deficient embryos for *emx1* (purple) and *smyhc1* (red). **(D)** Quantification of *emx1* domain length. **(E)** WISH (lateral view) of 28 ss WT, *mecom* deficient, and *mecom* deficient embryos co-injected with *emx1* capped mRNA (*emx1* cRNA rescue) for the DL segment marker *slc12a3* (purple). (**A, C, E**) Blue boxes demarcate domains of pronephros expression. Scale bar = 100 μm. **(F)** Quantification of DL length for WT, *mecom* deficient, and *emx1* cRNA rescue embryos. **(G)** Quantification of phenotype percentages for **(F)**, categorized as WT, decreased, or other, where the latter include those developmentally delayed or too dysmorphic to measure. **p < 0.01; ***p < 0.001; N.S. = not significant. Error bars represent standard error.

First, we assessed the relationship between *emx1* and *mecom*. We performed WISH in *emx1* deficient embryos to examine *mecom* expression, and observed no change compared to WT controls (Fig. 6A,B). Conversely, *mecom* deficient embryos had a significantly shorter domain of *emx1* expression in the distal pronephros (Fig. 6C,D), suggesting that *mecom* is upstream of *emx1*. To test this, rescue studies were performed to determine if *emx1* expression could restore DL segment length in the context of *mecom* loss of function. Provision of *emx1* capped mRNA (cRNA) was sufficient to rescue DL segment length in approximately 50% of *mecom* deficient embryos (Fig. 6E–G). Conversely, provision of *mecom* cRNA was not sufficient to rescue the DL in *emx1* morphants (Fig. S7). These results lead us to conclude that *mecom* acts upstream of *emx1*, and suggest that *emx1* is a critical effector of Mecom, either directly or indirectly, during pronephros development.

**Figure 7:**
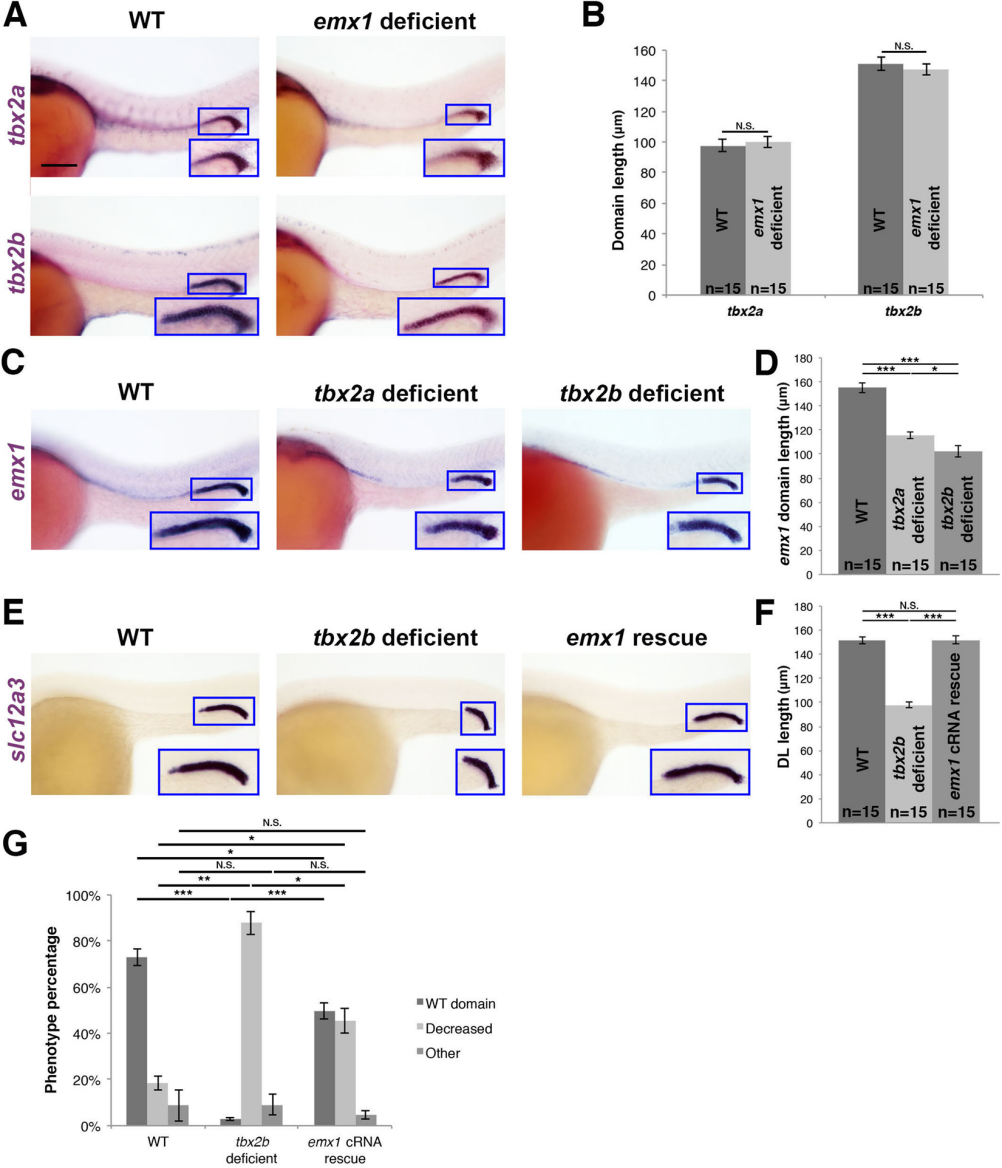
*tbx2b* acts upstream of *emx1* to regulate DL segment development. (**A**) WISH (lateral view) of 28 ss WT and *emx1* deficient embryos for *tbx2a* or *tbx2b* (purple). **(B)** Quantification of *tbx2a* and *tbx2b* domain length. **(C)** WISH (lateral view) of 28 ss WT, *tbx2a* deficient, and *tbx2b deficient* embryos for *emx1* (purple). **(D)** Quantification of *emx1* domain length. **(E)** WISH (lateral view) of 28 ss WT, *tbx2b* deficient, and *tbx2b* deficient embryos co-injected with *emx1* capped mRNA (*emx1* cRNA rescue) for the DL segment marker *slc12a3* (purple). **(A**,**C**,**E)** Blue boxes demarcate domains of pronephros expression. Scale bar = 100 μm. **(F)** Quantification of DL length in WT, *tbx2b* deficient, and *emx1* cRNA rescue embryos. **(G)** Quantification of phenotype percentages for **(F)**, categorized as WT, decreased, or other, where the latter include those developmentally delayed or too dysmorphic to measure. *p < 0.05; **p < 0.01; ***p < 0.001; N.S. = not significant. Error bars represent standard error.

Next, we explored the relationship between *tbx2a/b* and *emx1*. Using WISH, we found that there was no change in the spatial expression domain of either *tbx2a* or *tbx2b* in *emx1* deficient embryos (Fig. 7A,B). Thus, these *tbx2* genes are expressed ectopically in the expanded DE of *emx1* morphants, but to no phenotypic consequence, consistent with the absence of effects on DE fate in previous *tbx2a/b* gain or loss of function studies^23^. Conversely, the *emx1* expression domain was shortened in *tbx2a* and *tbx2b* deficient embryos (Fig. 7C,D). These results suggested that *emx1* acts downstream of these *tbx2* genes. As *tbx2a* acts upstream of *tbx2b* during DL development and *tbx2a* overexpression is associated with a greater incidence of dysmorphic gastrulation^23^, we therefore tested whether *emx1* cRNA could rescue the DL in the absence of *tbx2b* function. Interestingly, *emx1* cRNA was sufficient to rescue DL fate in approximately 50% of *tbx2b* deficient embryos (Fig. 7E–G). Conversely, provision of *tbx2b* cRNA was not sufficient to rescue the DL in *emx1* morphants (Fig. S7). In sum, we concluded that *emx1* acts downstream of *tbx2b* during DL development.

**Figure 8:**
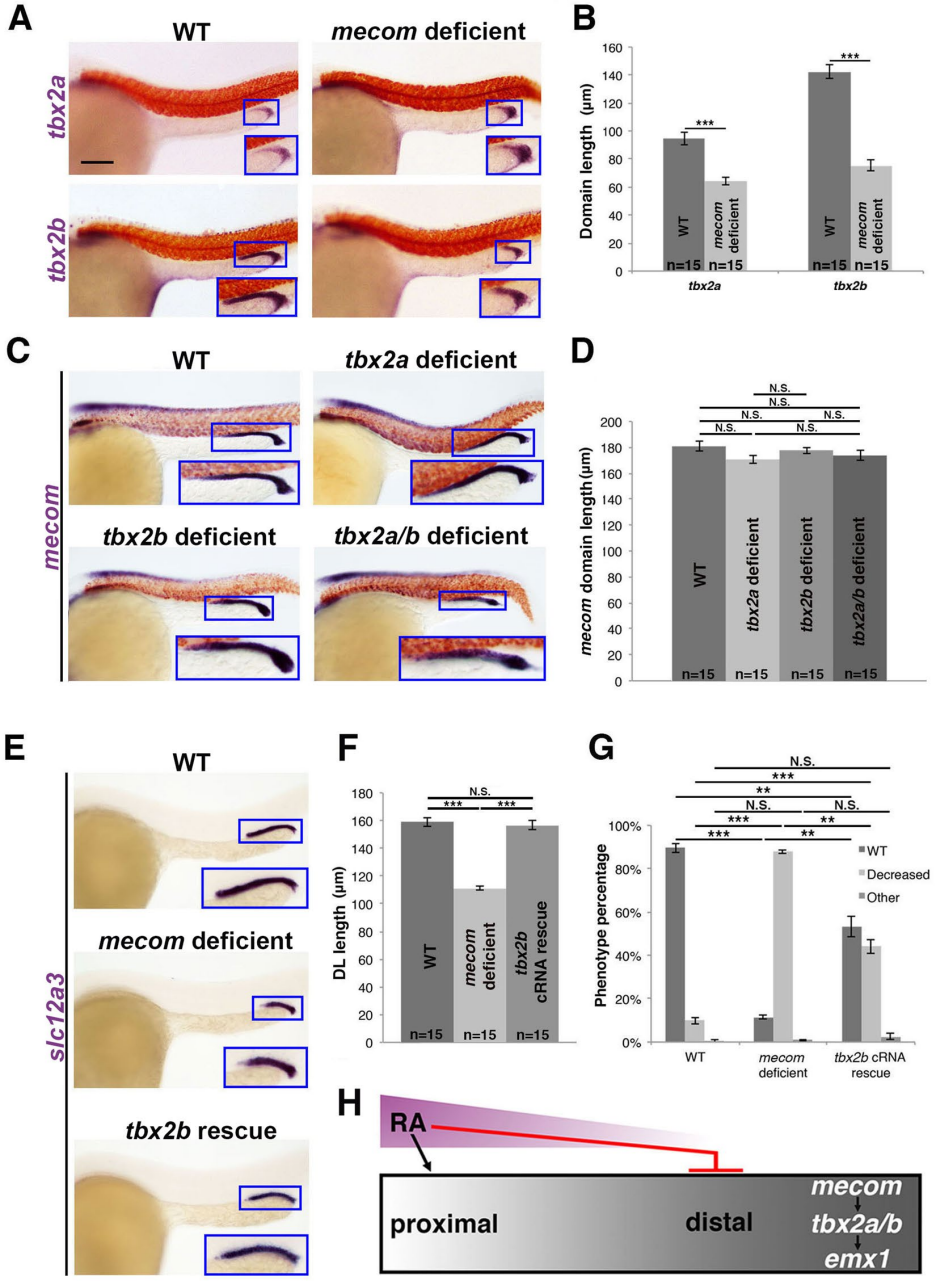
*mecom* functions upstream of *tbx2b* during DL segment formation (**A)** WISH (lateral view) of 28 ss WT and *mecom* deficient embryos for *tbx2a* or *tbx2b* (purple). **(B)** Quantification of *tbx2a* and *tbx2b* domain length. **(C)** WISH (lateral view) of 28 ss WT, *tbx2a* deficient, *tbx2b* deficient, and *tbx2a/b deficient* embryos for *mecom* (purple). **(D)** Quantification of *mecom* domain length. **(E)** WISH (lateral view) of 28 ss WT, *mecom* deficient, and *mecom* deficient injected with *tbx2b* capped mRNA (*tbx2b* cRNA rescue) for *slc12a3* (purple). **(A**,**C**,**E)** Blue boxes demarcate domains of pronephros expression. Scale bar = 100 μm. **(F)** Quantification of DL length in WT, *mecom* deficient, and *tbx2b* cRNA rescue embryos. **(G)** Quantification of phenotype percentages for **(F)**, categorized as WT, decreased, or other, where the latter include those developmentally delayed or too dysmorphic to measure. **p < 0.01; ***p < 0.001; N.S. = not significant. **(H)** RA promotes proximal segment development while inhibiting distal segments. *mecom* promotes distal segment fates as well as expression of *tbx2a/b*, which in turn promotes expression of *emx1*. *mecom* may also promote *emx1* through other targets than the *tbx2* genes (not shown). Error bars represent standard error.

In light of our findings that positioned both *mecom* and *tbx2b* as upstream of *emx1*, we examined the *mecom-tbx2b* relationship. *mecom* deficient embryos had reduced expression of *tbx2b* in the developing nephron, as well as *tbx2a* (Fig. 8A,B). Reciprocally, the *mecom* expression domain was unchanged in *tbx2a* or *tbx2b* deficient embryos (Fig. 8C,D), consistent with its pan-distal expression domain during late somitogenesis. Based on these results, we hypothesized that *mecom* is upstream relative to the *tbx2* genes in distal pronephros ontogeny. Given the limitations in *tbx2a* overexpression, we tested the ability of *tbx2b* cRNA to rescue DL segment fate the context of *mecom* loss of function. Consistent with the notion that *mecom* acts upstream of *tbx2b* either directly or indirectly during DL development, *tbx2b* cRNA was sufficient to rescue DL length in just over 50% of *mecom* deficient embryos (Fig. 8E,F,G). Taken together, we concluded that a genetic cascade of *mecom*, followed by *tbx2a/2b*, regulates *emx1* expression to control DL formation (Fig. 8H). Additionally, *mecom* may also regulate *emx1* through targets other than *tbx2b*, and all of these genetic interactions may be direct or indirect.

### *emx1* deficient nephrons show alterations in distal expression patterns of the *irx3b*, *irx1a*, and *sim1a* transcription factors

Following our studies analyzing the relationships between *mecom*, *tbx2a/b* and *emx1* that provided insight into the molecular changes underlying the DL phenotype in *emx1* deficient embryos, we turned our attention to elucidating the basis of the DE and CS phenotypes caused by *emx1* loss of function. Several factors have been associated with development of the DE. In the frog pronephros, *Irx3* is requisite for DE fate and abrogation of *Irx3* leads to loss of *Irx1* as well, leading to the hypothesis that an Irx3-mediated gene network controls the DE lineage^21^. Conservation of this pathway has been suggested by analysis of *irx3b* function in the zebrafish pronephros, where *irx3b* deficiency similarly caused loss of the DE lineage^20,45^. While *irx3b* transcripts are expressed in both the PST and DE segments^20^, expression of *irx1a* has been attributed to the approximate location of only the DE segment in zebrafish^46^.

**Figure 9:**
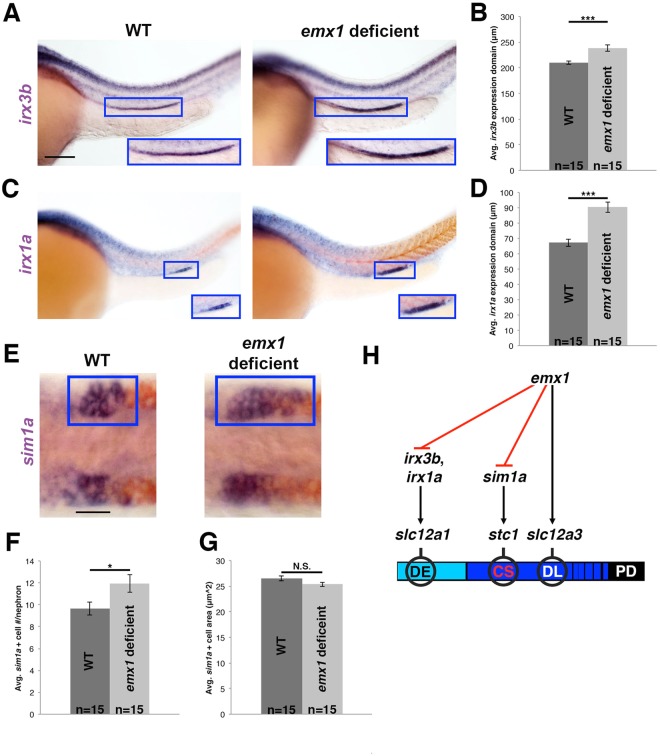
*emx1* deficiency results in increased *irx3b*, *irx1a*, and *sim1a* expression domains. **(A**) WISH (lateral view) of 28 ss WT and *emx1* deficient embryos for *irx3b* (purple). (**B**) Quantification of *irx3b* domain length. **(C)** WISH (lateral view) of 28 ss WT and *emx1* deficient embryos for *irx1a* (purple) and *smyhc1* (red). (**A**,**C**) Blue boxes demarcate domains of pronephros expression. Scale bar = 100 μm. (**D**) Quantification of *irx1a* domain length. (**E**) WISH (dorsal view) of 28 ss WT and *emx1* deficient embryos for *sim1a* (purple) and *slc12a3* (red). Blue boxes demarcate a single CS territory while the contralateral side is unlabeled. Scale bar = 20 μm. (**F**,**G**) Quantification of *sim1a*^+^ cell number and size. *p < 0.05; ***p < 0.001; N.S. = not significant. Error bars represent standard error. (**H**) *emx1* restricts formation of the DE by inhibiting expression of *irx3b* and *irx1a*, restricts the CS anlage by inhibiting *sim1a* expression, and promotes DL fate (*slc12a3*^+^) by regulating unknown targets.

To investigate the relationship between *emx1* with these *irx* genes, WISH studies were performed in WT and *emx1* deficient embryos. Interestingly, we observed that the expression domains of both *irx3b* and *irx1a* were distally expanded in length in *emx1* deficient embryos, and that these changes were significant compared to WT controls (Fig. 9A–D). These changes correlated with the expanded DE segment size that occurs in *emx1* loss of function, and suggest the intriguing hypothesis that *emx1* negatively regulates DE fate by repressing *irx3b* and *irx1a*, either directly or indirectly, in renal progenitors. Alternatively, the changes in *irx* expression domains may simply reflect the changes in segment fates in *emx1* deficient embryos.

With respect to the CS, expression of the transcription factor *single-minded family bHLH transcription factor 1a* (*sim1a*) is necessary and sufficient for CS fate choice^25,47^. Thus, we next examined the pattern of *sim1a* expression in *emx1* deficient embryos at the 28 ss, when CS anlage precursors can be visualized as bilateral clusters of cells located just dorsal to the pronephros. *emx1* deficient embryos had significantly increased numbers of *sim1a*^+^ cells, which had similar dimensions as WT cells (Fig. 9E–G). These results may indicate that *emx1* regulates the CS by repressing *sim1a* expression, either directly or indirectly, or simply that the changes in *sim1a* expression reflect the altered CS lineage in *emx1* deficient embryos. Taken together, we propose a testable working model where *emx1* is responsible for regulating the DE by controlling *irx3b* and *irx1a*, and for regulating the CS by controlling *sim1a* expression, which can be investigated further in future studies (Fig. 9H).

## Discussion

Nephron segmentation is an important developmental process that patterns the renal functional units into structures with spatially distinct functional domains. The precise order of segments, along with their scale and cell type composition, is essential for proper kidney function. Disruptions of segment formation, as well as damage to segment populations, are associated with various forms of kidney disease^48^. There have been exciting advances in our knowledge about nephrogenesis^49^, including the expression profiles of nephron segments during development, but there is still an incomplete understanding of the mechanisms that elaborate segmental fate decisions. Further advancements in the cultivation of renal cell types from pluripotent cell sources can be facilitated by the continued discovery of nephrogenesis processes^50^.

Here, we have shown for the first time that *emx1* is important for proper development of distal nephron segments in the zebrafish embryonic kidney. Our results indicate that *emx1* is necessary for promoting DL fate and inhibiting DE fate, where *emx1* acts in a genetic cascade downstream of *mecom* and *tbx2b*. We have also ascertained the relationship between *emx1* and several known segmentation regulators in the pronephros. This led us to assemble an integrated hierarchy of the upstream factors that regulate *emx1* (Fig. 8H), and to identify several components situated downstream of *emx1* (Fig. 9H). Additional studies are needed to delineate whether these relationships involve direct or indirect interactions. The identification of a putative EMX1 binding site or consensus sequence (TAATTANNTAATTA)^51^ will be useful in identifying direct downstream target candidates of *emx1*.

Further work is also needed to situate *emx1* activity with respect to other known segmentation pathways, such as Notch signaling and prostaglandin signaling. Notch signaling mitigates the choice between transporter fate and multiciliated cell (MCC) fate in several segment domains of the pronephros^52,53^. Because *emx1* deficiency expands the DE, which is one of the regions where MCCs are found, it is possible that *emx1* activity articulates with Notch to influence MCC genesis. With regard to segmentation, prostaglandin signaling is essential to control the balance of DE and DL transporter cell fates in the developing zebrafish pronephros^54^. Interestingly, the abrogation of prostaglandin biosynthesis or inhibition of receptor expression leads to an expanded DE and reduced DL^54^, which closely mimic how the *emx1* loss of function impacts distal pronephros segmentation. Given these similarities, is reasonable to speculate that *emx1* mitigates DE and DL fate wholly or in part through an interaction with prostaglandin signaling. Some immediate possibilities to consider are that Emx1 regulates the expression of prostaglandin synthesis enzymes or receptors within the renal progenitors or the neighboring tissues. Alternatively, *emx1* may act as a key transcriptional target of prostaglandin signaling. Further studies to assess prostaglandin signaling activity in *emx1* deficient embryos could be employed to ascertain the relationships between these pronephros development components.

Additionally, there may be currently unknown molecular components relevant to *emx1* activity during nephron segmentation. For example, during mammalian brain development, Hedgehog signaling has been placed upstream of Emx1^55^. Specifically, Gli3, a transcriptional regulator that is induced by Hedgehog signaling in the murine brain, is essential for *Emx1* expression^55^. Despite the need for more studies to appreciate the relationships between *emx1* and other pathways within the kidney, the genetic network maps assembled in this work provide a substantive initial framework for continued segmentation studies in the zebrafish pronephros.

The present study is the first report to attribute roles for *emx1* during mesoderm development. In contrast, *ems/Emx1* have long been known for their associations with the formation of the nervous system in the fly and mammal, respectively^30^. *ems/Emx1* control specification of the neuroectoderm early in embryogenesis, and they play subsequent roles during assembly of the olfactory sensory system and visual system where they control neuron development^30,56^. Exciting recent proteomics approaches have begun to elucidate the molecular features associated with Emx1 loss of functionin neurogenesis^57^, which may provide insights into EMX1 activities that will be relevant in the context of other tissues as well. Among EMX1 interacting partners is *WDR11*, a WD domain-containing gene, mutations of which have been linked to Idopathic Hypogonadotropic Hypogonadism and Kallmann Syndrome (IHH/KS) in humans^58^. Male patients with KS display unilateral renal agenesis with a ~30% frequency^59–61^. The EMX1/WDR11 association warrants further investigation both in light of our findings, and given the expression of Emx1 in the developing mammalian kidney.

In contrast to *Emx1*, its family member *Emx2* is well-established as essential for mammalian kidney development. In the developing murine kidney, for example, *Emx2* is expressed in the pronephros primordium, mesonephros tubules and later the metanephros^62^. Within the latter, *Emx2* is expressed in the invading ureteric bud and branches, along with the renal vesicles and comma shaped bodies that represent early nephron structures^62^. GUDMAP microarray data from E15 murine kidney have similarly shown *Emx2* expression in the renal vesicles, as well as the proximal tubules, loop of Henle anlage, and collecting ducts at E15, which also mirrors expression of *Emx1*^36^. Knockout of *Emx2* causes severe disruptions of urogenital system formation, where the metanephric kidneys fail to develop^63^. Most recently, expression of *Emx2* along with *Hnf1b*, *Hnf4a* and *Pax8* was found to be sufficient to convert both mouse and human fibroblasts into induced renal tubular epithelial cells^64^. Interestingly, the same study reported the expression domains of both *emx2* and *emx1* in the *Xenopus* pronephros, where these factors initially co-localize in the nephron tubule region and then become restricted to the tubule and duct, respectively^64^. Similarly in the zebrafish pronephros, *emx2* is expressed in the proximal renal progenitors^27^, while *emx1* is expressed proximally and then becomes restricted to the distal renal progenitor domain, as illustrated in the present work and previous studies^27^. Taken together, there are clear differences across vertebrates with regard to the expression of *emx1/Emx1* in developing nephrons, where lower vertebrates exclude *emx1* expression from the proximal tubule during pronephros ontogeny, which is appears to be distinct compared to nephrogenesis in the mammalian metanephros. Analysis with dual *in situ* hybridization or immunofluorescence would be useful to further discern the spatiotemporal dynamics and respective expression domains of *Emx1/Emx2* during nephrogenesis in the metanephros. Functional studies are also needed to ascertain the role(s) of *emx2* in pronephros development, and the relationship between *emx1* and *emx2*.

Here, our current study has established a framework of the transcriptional network within which *emx1* functions to regulate zebrafish nephron segment development. As the conserved segment features of zebrafish and mammalian nephrons^65^ lend credence to the notion that insights about segmentation mechanisms in the teleost pronephros can be used as an entrée to help further our understanding of nephrogenesis in other vertebrates, the newly discovered functions of *emx1* provide a novel rationale for testing the roles of its ortholog during mammalian nephrogenesis. Continuing to elucidate how nephron segmentation occurs is relevant to deciphering the basis of congenital kidney defects, and will be instrumental in improving approaches to combat renal disease with regenerative medicine.

## Methods

### Zebrafish husbandry and ethics statement

Zebrafish were housed and cared for in the Center for Zebrafish Research at the University of Notre Dame Freimann Life Science Center, where the Institutional Animal Care and Use Committee (IACUC) approved the experiments documented here under protocol numbers 13–021 and 16–025. All methods were carried out in accordance with relevant guidelines and regulations. Tübingen strain WT zebrafish were used and staged as described^66^. Embryos were incubated at 28 °C in E3 medium^67^.

### Morpholinos

Morpholinos were purchased from Gene Tools, LLC (Philomath, OR), solubilized with DNase/RNase free water, and stored at −20 °C. The *emx1* ATG-MO 5′-CCGTTGCCGAGAACATTGTCCGTGA-3′ targets the start site^42^. The combination of *emx1* splice-blocking MOs (*emx1* SB-MO), *emx1* SD 5′-AATACTTACCTTGAAACCGATGTCC-3′ and *emx1* SA 5′-GAGACATCATTACCTAAGATATAAC-3′ were designed to bind the splice donor and acceptor sites of exon 1 and exon 2, respectively. WT embryos were injected at the 1-cell stage with 1 nL of 133 μM *emx1* ATB-MO or 400 μM *emx1* SB-MO. The *tbx2* morpholinos were^23^: MO1-tbx2a, which targets *tbx2a* 5′-ATCGGTGCATCCAAAAAGCCAGAT-3′; MO1-tbx2b, which targets *tbx2b* 5′-CCTGTAAAAACTGGATCTCTCATCG-3′; MO1-tbx2, which targets both *tbx2a/b*, 5′-AAAACTGGATCTCTCATCGGTGCAT-3′. The *mecom* morpholinos were^22^: MO3-mecom, 5′-CTGAGTGACTTACATATGAAGGGCT-3′ and MO4-mecom, 5′-TTGTGGCAGACCTCACCTAGAGACA-3′, which target splicing of *mecom*.

### Reverse transcription PCR

RNA was extracted from WT embryos and *emx1* SB-MO morphants at 24 hpf with Trizol reagent (Invitrogen) according to manufacturer instructions. RT-PCR was performed using the SuperScript First-Strand Synthesis System (Invitrogen)^26^. To confirm aberrant splicing of *emx1* intron 1 in *emx1* SB-MO morphant embryos, PCR primers were specifically designed to amplify a region corresponding to 620 bp of sequence that contains both the 3′-end of *emx1* exon 1 and the 5′-end of *emx1* intron 1 (forward: 5′-AACATCGTGAACCACTGAATTTCTACCCTTGGGTCCTAAGGAACAGGTTTTTTGGACATC-3′ and reverse 5′- CAATTGACTTCCATAATAAGAAAACAATAGCCTGCTGCTGGAAGTCATTGGTTCCAGGTTTCTAGC-3′).

### cRNA synthesis and rescue experiments

An *emx1* pCS2 plasmid was first constructed by obtaining an *emx1* clone containing the *emx1* ORF flanked by an EcoRI restriction site at the 5′ end and an XhoI restriction site at 3′ (Clone ID: ODa00797C) (GenScript). The *emx1* ORF sequenced was then excised from the *emx1* clone and directionally cloned into the pCS2 vector to be used for cRNA synthesis. Synthetic *emx1* cDNA was synthesized from the *emx1* pCS2 plasmid using an sp6 mMessage Machine kit (Ambion) after linearization of the plasmid template. *mecom* and *tbx2b* cRNA was synthesized as described^22,23^. To perform rescue experiments, 1-cell stage embryos were injected with 12–15 pg *emx1* cRNA^42^, 25 pg *mecom* cRNA^22^ or 400 pg *tbx2b* cRNA^23^ along with the appropriate morpholino(s). For all rescue studies, replicate group sizes were a minimum of 30 embryos, and typically ranged between 40–60 embryos for each cohort.

### WISH, FISH, IF and acridine orange staining

For gene and protein expression analyses, embryos were fixed in 4% paraformaldehyde/1X PBST and stored in 100% methanol at −20 °C. Biological triplicates of all genetic conditions were analyzed for expression of each marker, where replicates consisted of 30–40 embryos. WISH^68,69,70^ and FISH^71^ were performed as described. Antisense probes for *smyhc1*, *deltaC*, *cdh17*, *slc4a4a*, *clcnk*, *slc20a1a*, *trmp7*, *slc12a1*, *stc1*, *slc12a3*, *gata3*, *emx1*, *irx3b*, *sim1a*, and *mecom* were generated using IMAGE clone template plasmids^20,25^. Antisense probes for *emx1* and *irx1a* were generated using IMAGE clones 9001097 and 9039039, respectively. Probe for *emx1* was transcribed from PCR-generated DNA templates amplified with primers (forward: 5′-TTTATGGACACCACTGGATCG-3′ and reverse: 5′-AATTAACCCTCACTAAAGGGTTCTTCTTTTGTGTGCACTCCGGG-3′). Probe for *irx1a* was transcribed from the PCR-generated DNA templates with primers (forward: 5′-ATGTCTTTCCCCCAGCTGGGCTACCCG-3′ and reverse: 5′-AATTAACCCTCACTAAAGGGTCAAGCGGAGGA-3′. Immunofluorescence studies to detect pH3 and Caspase-3, and acridine orange treatment to detect apoptotic cells were performed as described^71,72,73^.

### Chemical treatments

RA and DEAB (Sigma-Aldrich) were dissolved in 100% DMSO to make a 1 M stock solution and aliquots were stored at −80 °C in the dark^74^. For drug treatments, embryos were incubated in the dark from 60% epiboly to the 15 ss in either 1 × 10^−7^ M RA/DMSO, 1.6 × 10^−5^ M DEAB/DMSO, or 1.6 × 10^−5^ M DMSO in E3 media^75^. Embryos were then washed 5 times with E3 to remove exogenous chemicals, left to develop at 28 °C until 24 hpf, after which they were euthanized and fixed. These chemical treatments were fully penetrant and produced consistent results over three replicates with n = 40–60 embryos per replicate analyzed for these studies.

### Measurements and statistics

For WISH and acridine orange-stained embryos, 5 embryos from both the WT and experimental group of each experiment were selected, photographed from a lateral view using a Nikon eclipse Ni with a DS-Fi2 camera, and had either the length of their pronephric stain measured in μm or the number of AO-positive cells counted using either the Nikon Elements software or FIJI. For *stc1* and *sim1a*-stained embryos dorsal photographs were taken and cell number and cell area we measured in μm^2^. For FISH and IF, 3 embryos from the WT and experimental groups were selected, from which lateral view z-stacks were acquired using a Nikon A1 confocal microscope. From the z-stack data sets, the parameters of segment lengths, IND, nuclei number, and tubule volume were measured using FIJI. For statistical analysis, t-tests were performed. For phenotype percentage comparisons, the proportion data were normalized via arcsine transformation before being compared using t-tests.

## Electronic supplementary material

Supplementary Information

## Data Availability

The data associated with this report are provided in the figures and supplemental figures.
