# Supplementary material for: Homeogene emx1 is required for nephron distal segment development in zebrafish
Source: Sci Rep. 2018 Dec 21;8:18038. doi: 10.1038/s41598-018-36061-4 (PMC6303317; doi:10.1038/s41598-018-36061-4)

Homeogene *emx1* is required for nephron distal segment development in zebrafish

Elvin E. Morales[1], Nicole Handa[1], Bridgette E. Drummond[1], Joseph M. Chambers[1], Amanda N. Marra[1], Amanda Addiego[1], and Rebecca A. Wingert[1,*]

[1]Department of Biological Sciences, Center for Stem Cells and Regenerative Medicine, Center for Zebrafish Research, University of Notre Dame, Notre Dame, IN 46556, USA

[*]Corresponding Author

Correspondence: Rebecca A. Wingert, Ph.D., Department of Biological Sciences, University of Notre Dame, 100 Galvin Life Sciences, Notre Dame, IN 46556 USA; Email: rwingert@nd.edu; Phone: (574)-631-0907; Fax: (574)-631-7413

SUPPLEMENTAL FIGURE LEGENDS

Supplemental Figure 1. *emx1* splice-blocking morpholino cocktail (SB-MO) prevents splicing of *emx1* intron 1. (A) Diagram depicting PCR reaction used to test the efficacy of the *emx1* SB-MO. *emx1* SD MO targets the splice donor site while *emx1* SA MO targets the splice acceptor site. F and R indicate the relative positions of the primers used in the PCR reaction. These primers will generate a 620 basepair (bp) PCR product only if *emx1* intron 1 is present in the cDNA templates synthesized through RT-PCR. Aberrant splicing of intron 1 can result in a truncated protein due to it containing a premature stop codon. **(B)** PCR products are shown for both WT and *emx1* SB-MO embryos. The desired PCR product could only be produced from cDNA of *emx1* deficient embryos but not from that of WT embryos, confirming the presence of *emx1* intron 1 in embryos injected with *emx1* SB-MO. *ef1α* serves as a positive control for the PCR reaction. **(C-J)** Quantification of phenotype percentages in WT, *emx1* deficient, and (where relevant) *emx1* cRNA rescue embryos. Phenotype categories are defined as WT, altered (increased for the DE, CS; decreased for the DL), and other (embryos that were developmentally delayed or too dysmorphic to measure). * = $p < 0.05$; ** = $p < 0.01$; *** = $p < 0.001$; N.S. = not significant. Error bars represent standard error.

Supplemental Figure 2. *emx1* deficiency does not affect PCT, PST, or PD segment development. (A-E) WISH panel depicting both WT and *emx1* deficient embryos stained for *slc4a4a* (pan-proximal domain)*, clcnk* (pan-distal domain)*, slc20a1a* (PCT), *trpm7* (PST), and *gata3* (PD) (purple) and *smyhc1* (red). Scale bar = 100 μm. **(F-J)** Quantifications of domain lengths. N.S. = not significant. Error bars represent standard error.

Supplemental Figure 3. *emx1* SB-MO phenotype is recapitulated by *emx1* start-site morpholino (*emx1* ATG-MO). (A-H) WISH panel of 28 ss WT and *emx1* ATG-MO injected embryos for pan-proximal, pan-distal, and individual segment markers (purple): *slc4a4a* for the pan-proximal domain, *clcnk* for the pan-distal domain, *slc20a1a* for the PCT, *trpm7* for the PST, *slc12a1* for the DE, *slc12a3* for the DL, *gata3* for the PD, and *stc1* for the CS. Scale bar for A - G = 100 μm; scale bar for H = 20 μm. **(I-O)** Quantifications of domain lengths. Quantification of *sim1a*$^{+}$ cell number **(P)** and size **(Q)**. *** = $p < 0.001$; N.S. = not significant. Error bars represent standard error.

Supplemental Figure 4. Penetrance of *emx1* ATG-MO in pronephric segments and overall domains recapitulates the *emx1* SB-MO phenotype distributions. (A-H) Quantification of phenotype percentages in WT and *emx1* ATG-MO injected embryos. Phenotype categories are defined as WT, altered (increased for the DE, CS; decreased for the DL), and other (developmentally delayed or too dysmorphic to measure). * = $p < 0.05$; ** = $p < 0.01$; N.S. = not significant. Error bars represent standard error.

Supplemental Figure 5. *emx1* deficiency does not result in any significant changes in cell death within the distal zebrafish pronephros, but does so within the head region. (A) WT and *emx1* deficient embryos dyed with acridine orange (AO) at the 28 ss to detect dying cells. Scale bar = 100 µm. Quantification of AO^+ cells counts in the distal pronephros **(B)** and the head **(C)** of WT and *emx1* deficient embryos. ** = $p < 0.01$; N.S. = not significant. Error bars represent standard error.

Supplemental Figure 6. Alterations in RA levels lead to expression domain alterations of the DL segment marker *slc12a3*. WISH panel (lateral view) of *slc12a3* expression (purple) in 28 ss embryos treated with dimethyl sulfoxide (DMSO) vehicle (left), all-trans retinoic acid (RA) (middle), or 4-diethylaminobenzaldehyde (DEAB) (right). Exogenous RA leads to a smaller domain of *slc12a3*, and DEAB leads to a smaller domain of *slc12a3* (indicated by blue box); scale bar = 100 µm.

Supplemental Figure 7. Overexpression of *mecom* or *tbx2b* is not sufficient to rescue DL segment development in *emx1* deficient embryos. (A) WISH (lateral view) of 28 ss WT (left, top) compared to *emx1* deficient embryo (left, bottom) compared to *emx1* deficient embryos injected with *tbx2b* cRNA (right, top) and *mecom* cRNA (right, bottom), stained with *slc12a3* (purple). Scale bar = 100 µm. **(B)** Quantification of DL domain length. *** = $p < 0.001$; N.S. = not significant. Error bars represent standard error.

Supplemental Figure 1

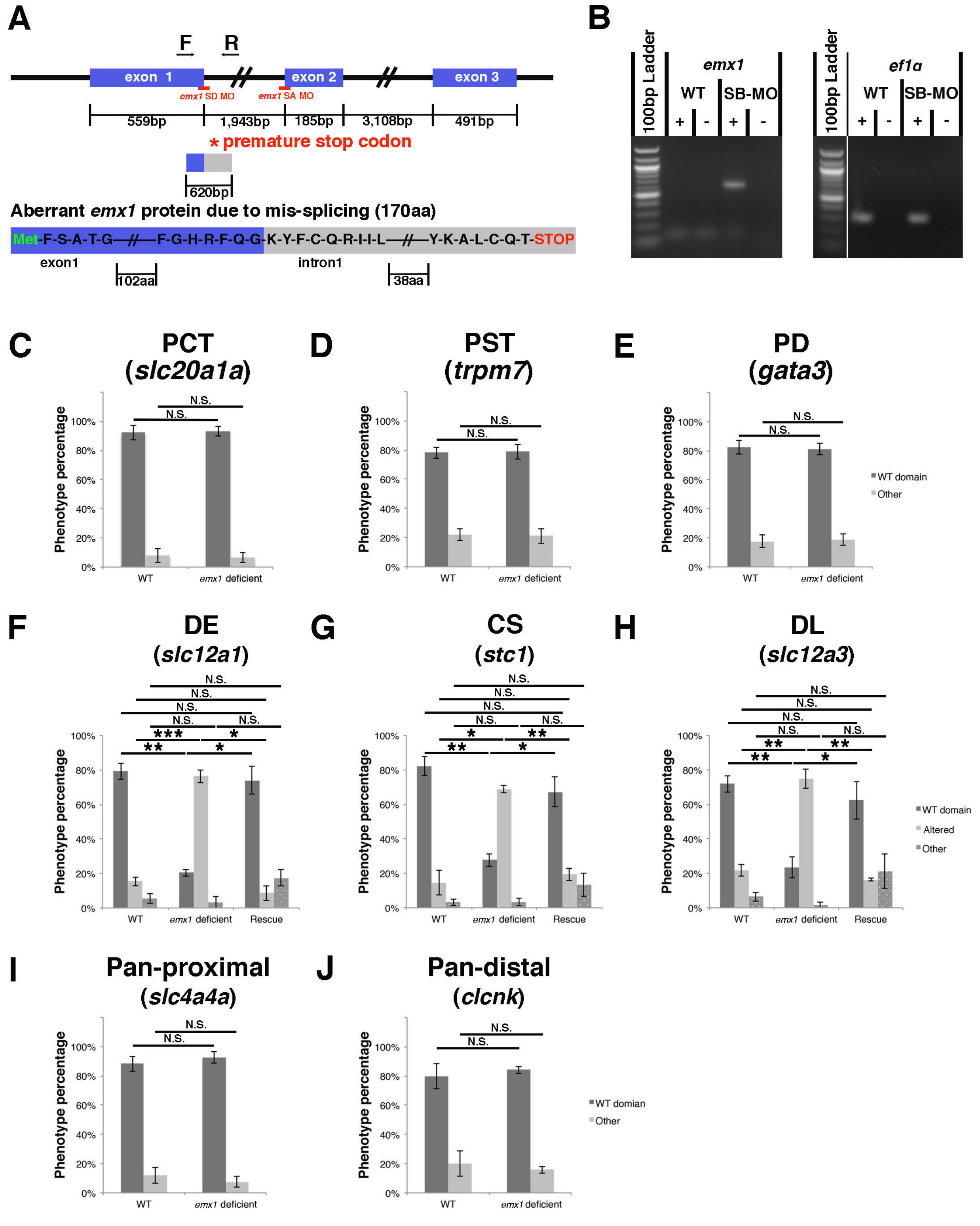

Supplemental Figure 2

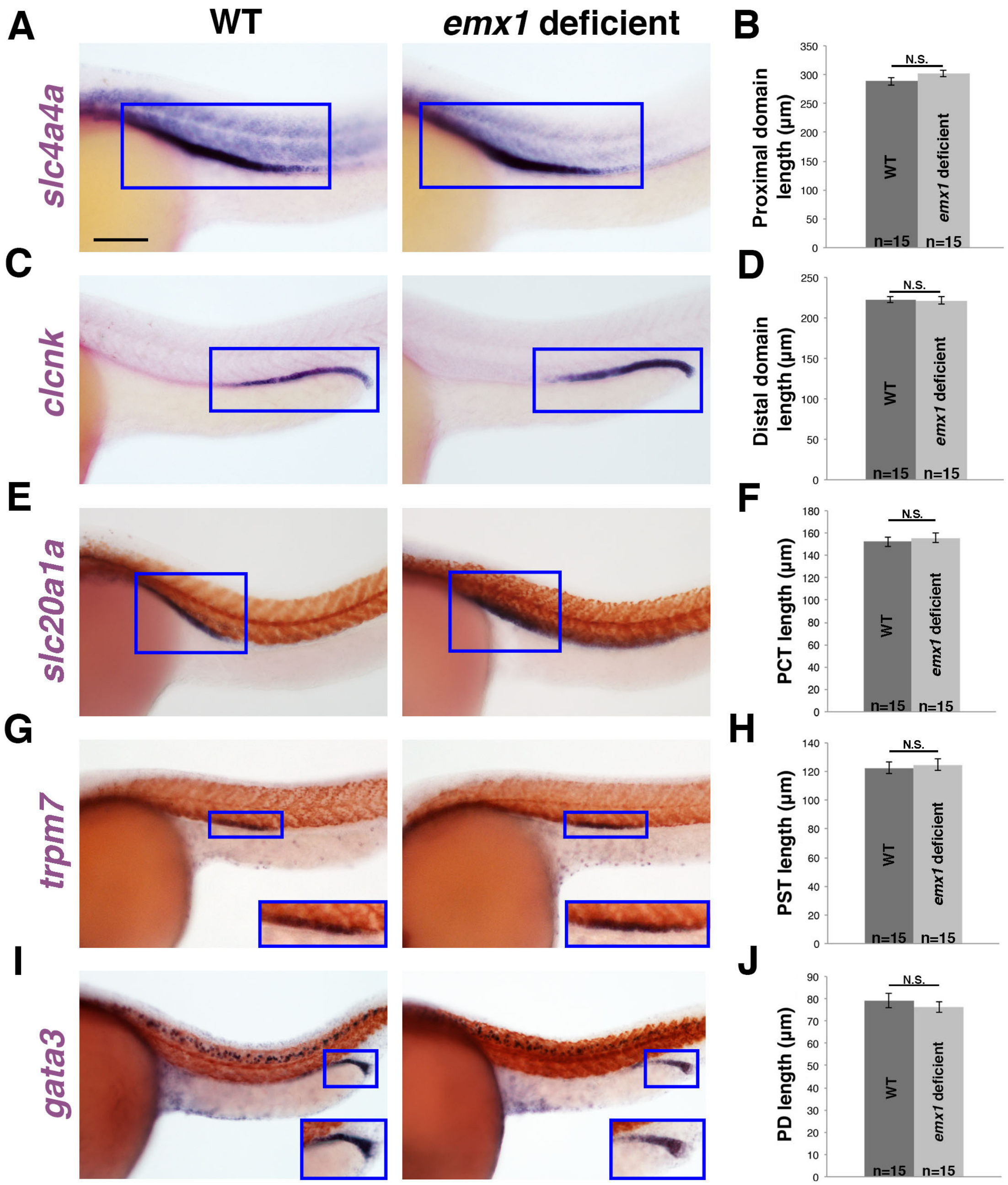

Supplemental Figure 3

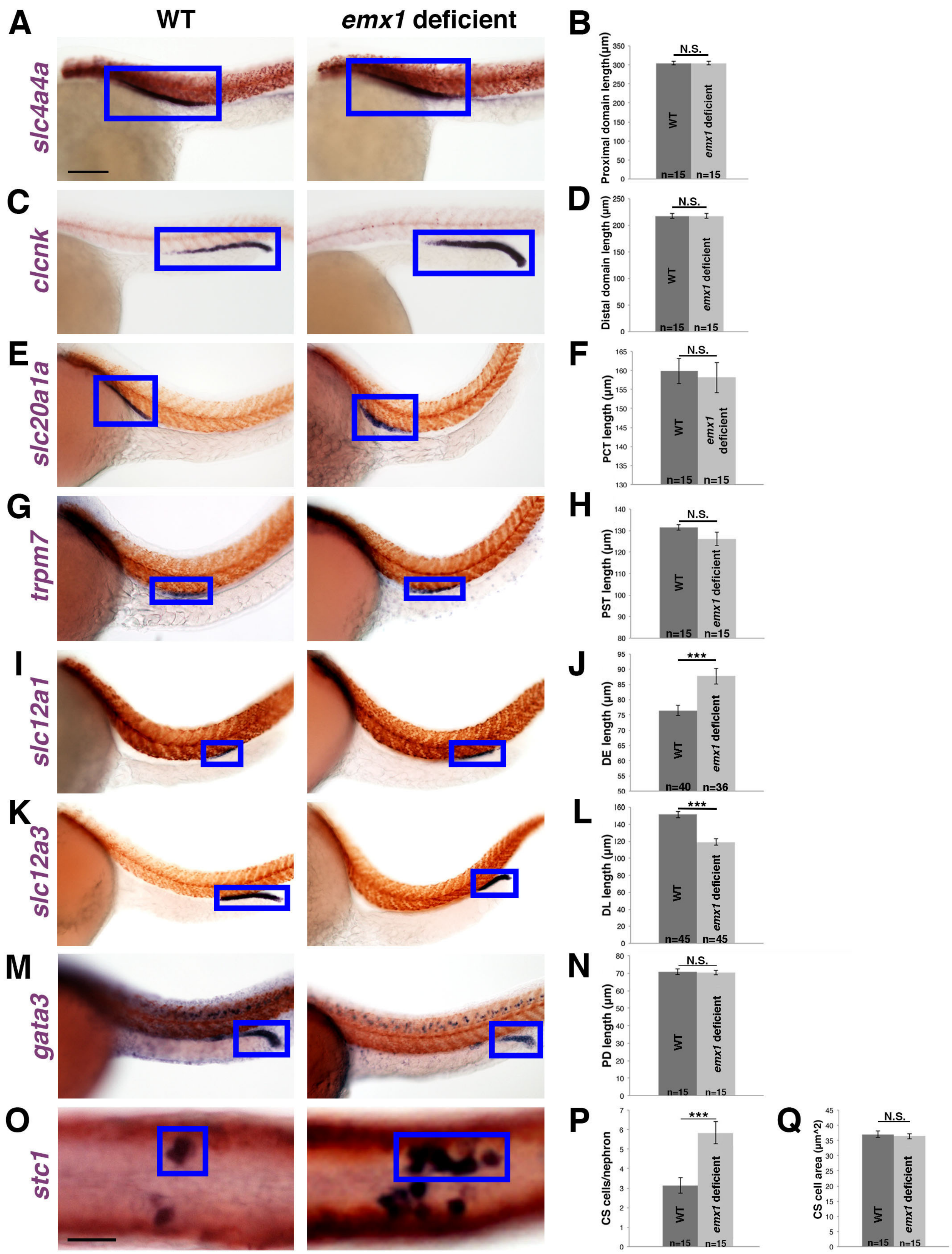

Supplemental Figure 4

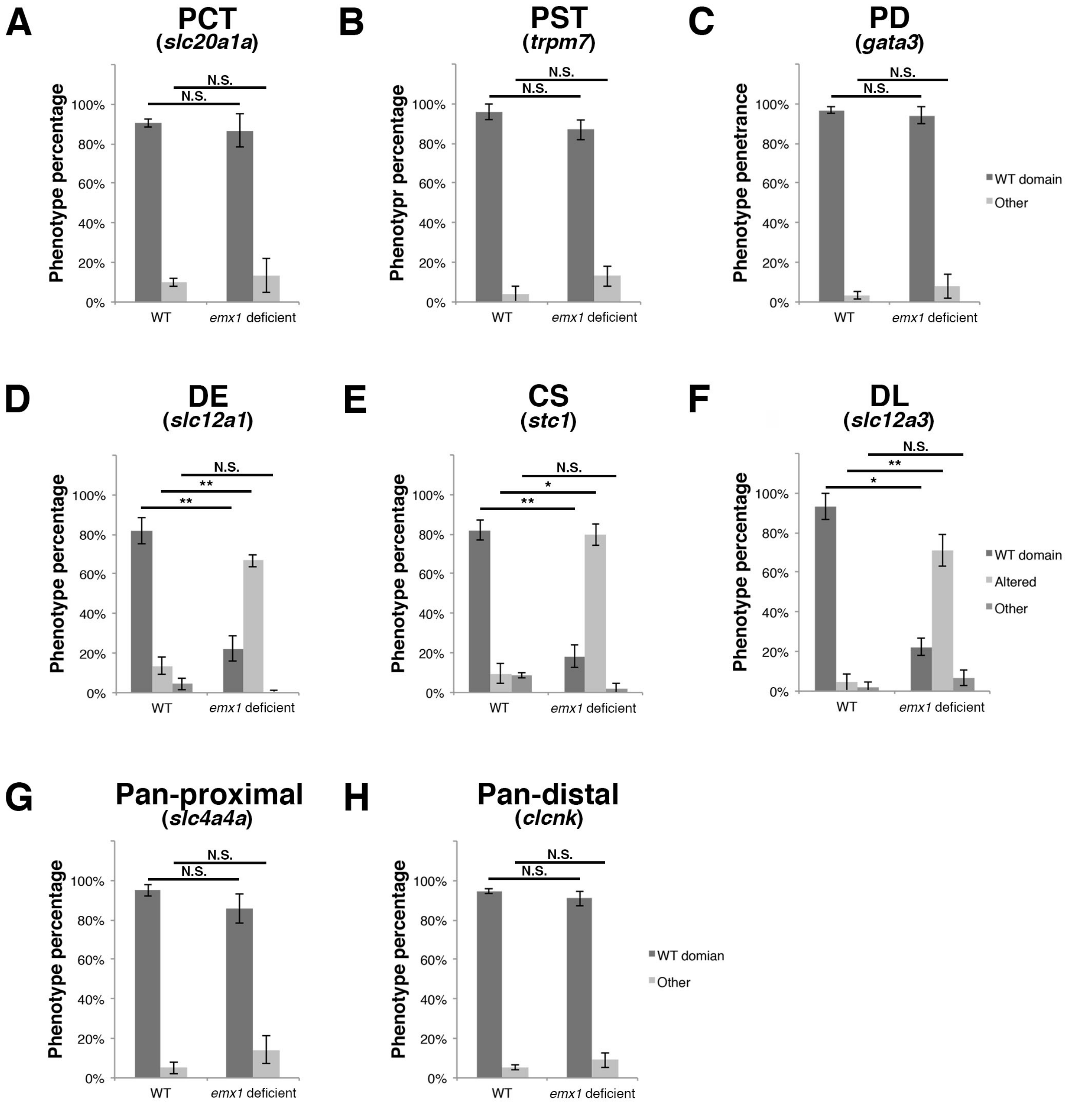

Supplemental Figure 5

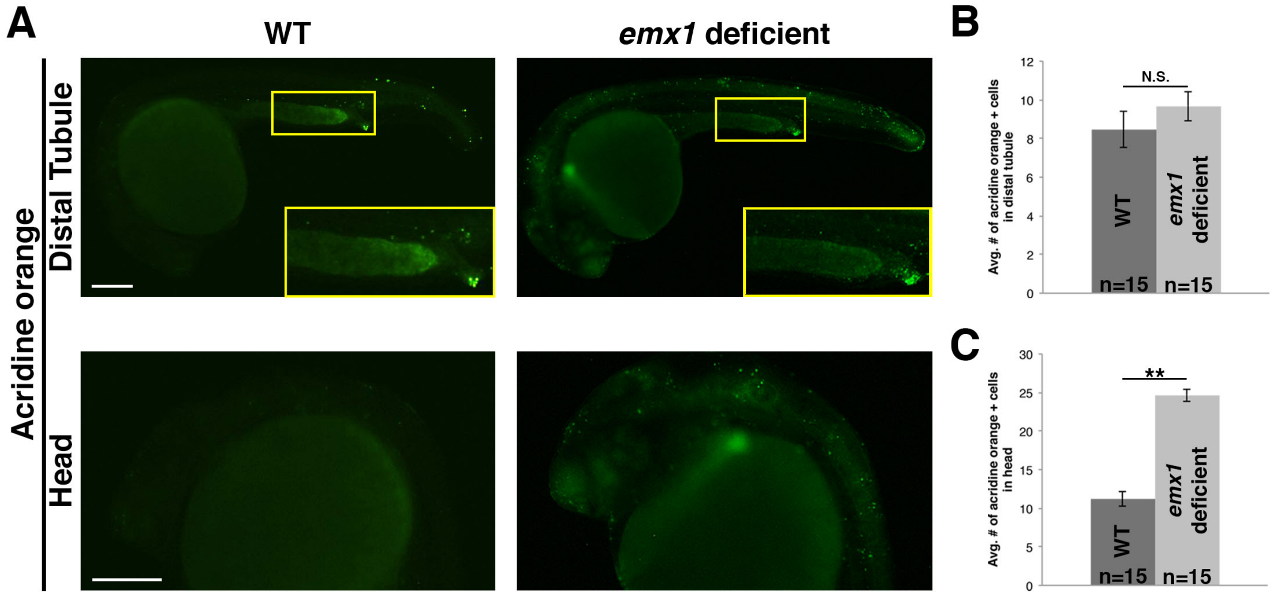

Supplemental Figure 6

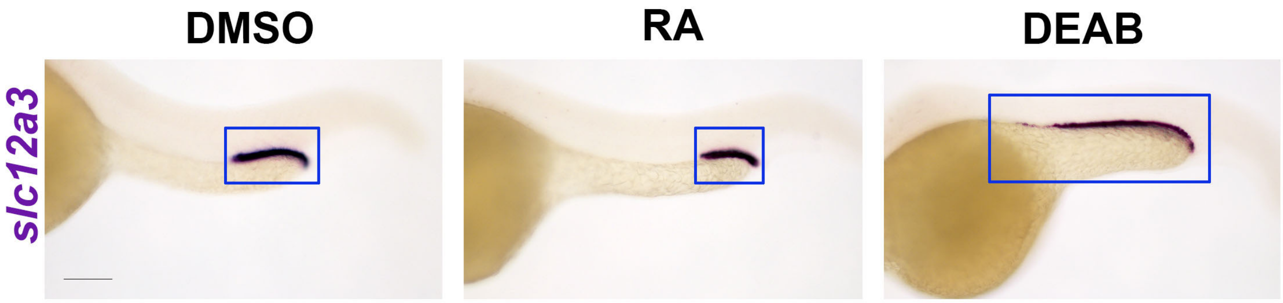

Supplemental Figure 7

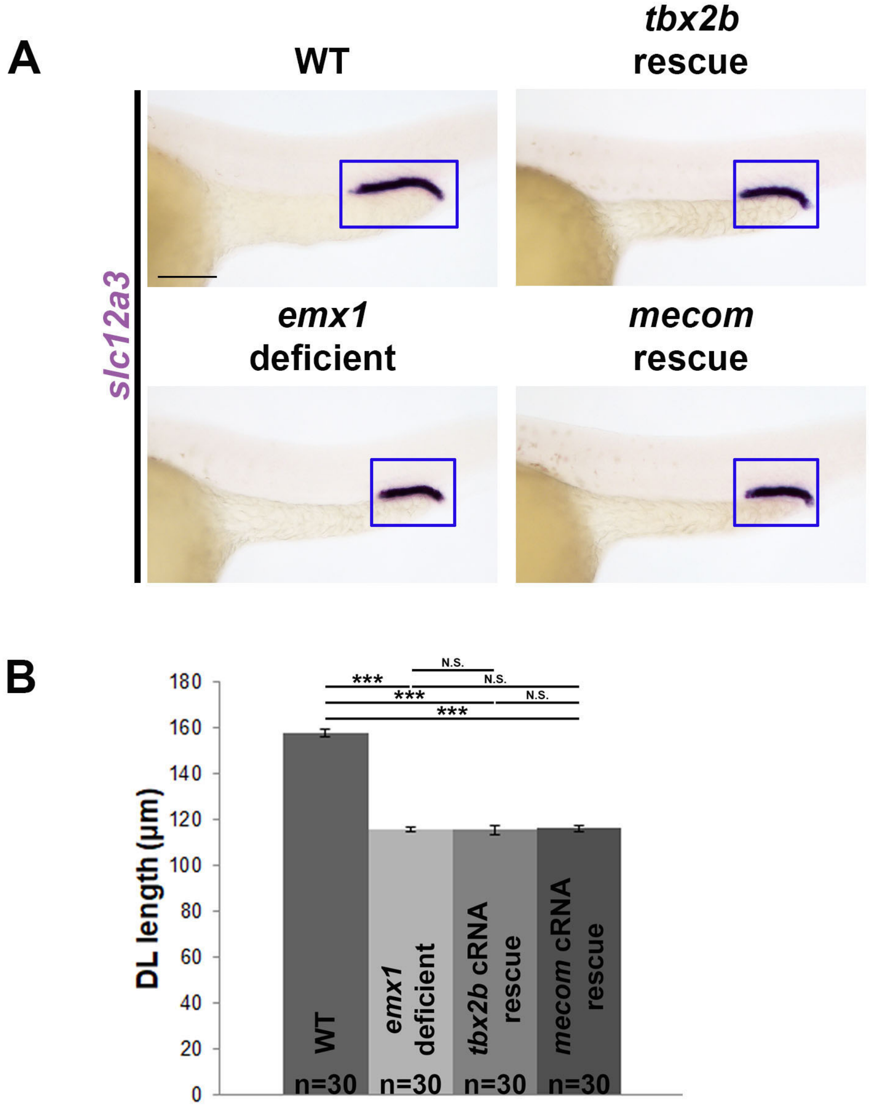

Supplement: Supplementary file 1 — Supplementary Information [file 41598_2018_36061_MOESM1_ESM.pdf]
